# A Comparative Study on the Faecal Bacterial Community and Potential Zoonotic Bacteria of Muskoxen (*Ovibos moschatus*) in Northeast Greenland, Northwest Greenland and Norway

**DOI:** 10.3390/microorganisms6030076

**Published:** 2018-07-25

**Authors:** Emilie U. Andersen-Ranberg, Christopher J. Barnes, Linett Rasmussen, Alejandro Salgado-Flores, Carsten Grøndahl, Jesper B. Mosbacher, Anders J. Hansen, Monica Alterskjær Sundset, Niels Martin Schmidt, Christian Sonne

**Affiliations:** 1 Department of Bioscience, Faculty of Science and Technology, Arctic Research Centre, Aarhus University, 4000 Roskilde, Denmark; jemo@bios.au.sk (J.B.M.); nms@bios.au.dk (N.M.S.); cs@bios.au.dk (C.S.); 2 Department of Veterinary Clinical and Animal Sciences, Faculty of Health and Medical Sciences, University of Copenhagen, 1870 Frederiksberg, Denmark; 3 Centre for GeoGenetics, Natural History Museum of Denmark, University of Copenhagen, 1350 Copenhagen, Denmark; c.barnes@snm.ku.dk (C.J.B.); linett.rasmussen@snm.ku.dk (L.R.); ajhansen@snm.ku.dk (A.J.H.); 4 Department of Arctic and Marine Biology, UiT—The Arctic University of Norway, 9037 Tromsø, Norway; alejandro.f.salgado@uit.no (A.S.-F.); monica.a.sundset@uit.no (M.A.S.); 5 Copenhagen Zoo, Centre for Zoo and Wild Animal Health, DK-2000 Frederiksberg, Denmark; cg@zoo.dk

**Keywords:** *Ovibos moschatus*, muskoxen, Arctic, zoonoses, 16S rRNA, metabarcoding, faecal bacterial community

## Abstract

Muskoxen (*Ovibos moschatus*) are ruminants adapted to a high-fibre diet. There is increasing interest in the role that gut microbes play in the digestion and utilization of these specialized diets but only limited data available on the gut microbiome of high-Arctic animals. In this study, we metabarcoded the 16S rRNA region of faecal samples from muskoxen of Northeast Greenland, Northwest Greenland and Norway, and quantified the effects of physiological and temporal factors on bacterial composition. We found significant effects of body mass, year of sampling and location on the gut bacterial communities of North East Greenland muskoxen. These effects were however dwarfed by the effects of location, emphasizing the importance of the local ecology on the gut bacterial community. Habitat alterations and rising temperatures may therefore have a considerable impact on muskoxen health and reproductive success. Moreover, muskoxen are hunted and consumed in Greenland, Canada and Alaska; therefore, this study also screened for potential zoonoses of food safety interest. A total of 13 potentially zoonotic genera were identified, including the genera *Erysipelothrix* and *Yersinia* implicated in recent mass die-offs of the muskoxen themselves.

## 1. Introduction

Only a few large terrestrial mammals have adapted to life in the high Arctic. The muskox (*Ovibos moschatus*) is the largest (150–300 kg) of only a handful of ruminants to do so and they roam areas of northern Canada, Northeast Greenland and Alaska. They have been further introduced to Norway, West Greenland, Wrangel Island and the Taimyr Peninsula [1]. Approximately 90,000 animals are estimated to exist in the wild in Alaska, Canada and Greenland but numbers are declining in Canada and Northeast Greenland [1,2]. Muskoxen mainly forage on graminoids and shrubs [3,4,5]. High Arctic summers are short and provide the muskoxen with energy rich pastures for a limited time [6]; the winter is long and offers limited forage that is high in fibres (lignocellulosic macromolecules) [5,7].

These fibres are the main source of energy for ruminants [8] and the degradation of fibre is mediated by (*i*) symbiotic anaerobic microbes producing short chain fatty acids, an essential source of energy for ruminants and (*ii*) long retention time in the rumen and hindgut providing enough time for efficient microbial fermentation [9,10]. Microbes present different abilities to ferment and process carbohydrates and perhaps unsurprisingly, diet is a key factor influencing the gut microbiota [11,12,13,14]. High Arctic muskoxen have evolved the specialized ability to utilize a diet rich in highly complex carbohydrates to survive the extreme conditions of the long winter of the high Arctic [15,16] and their digestive bacterial community plays an essential role in their survival [8]. Qi et al. [15] correspondingly found a high proportion of cellulolytic enzymes in the rumen of muskoxen. Though ruminal microbiomes have been extensively investigated [17], the sampling technique involved is not readily applicable to wild animals, unless post mortem. Faecal sampling on the other hand, represents a non-invasive and potentially low-stress procedure for sampling wild animals; and while a majority of the digestion is believed to occur in the rumen, it continues through the length of the intestinal tract including the hindgut.

The composition and regulation of muskoxen gut microbiota remains almost entirely unknown [15,16], with effects of location and subsequent variations in feed availability and climate, that are largely undetermined. It is also unknown whether the gut bacterial community varies within populations, with factors such as age, seasonal or annual fluctuations, sex and body mass never investigated. Given the likely importance of the gut microbial community in processing a high fibre diet, understanding the regulation of muskox gut microbial communities is extremely timely in light of the rapid changes occurring in the Artic regions. In agreement with this, Davidson et al., 2011 [18] (p. 6) proposed that “comparisons between Arctic fauna and relocated satellite populations at the edge of their climatic range (e.g., muskoxen imported to Norway from Greenland during the past century) can provide early warnings for potential threats to Arctic fauna resulting from alterations in the environment, such as climate change.”

Moreover, muskoxen are hunted and consumed in Northwest and West Greenland, certain areas of East Greenland as well as in Canada and Alaska. In West Greenland, by Kangerlussuaq, it is taking place as a semi-structured slaughter for commercial and herd control purposes. The potential transfer of zoonotic diseases by consumption or handling of muskoxen is a food safety and health issue that has not been investigated in a broad sense in Greenland and a desire for in depth investigations are iterated by the Inuit community as well as official bodies [19,20,21,22].

Here, we characterize the faecal bacterial composition via 16S rRNA sequencing of wild Northeast- and introduced Norwegian (Tromsø) muskoxen, with three main objectives: our first objective was to quantify the effects of physiological factors (sex, body mass) and temporal (year of sampling) effects on the gut bacterial composition of the Northeast Greenlandic muskoxen. The second objective was to quantify the effect of changing geographical location on the bacterial composition in muskoxen faeces, comparing populations from Norway and Northeast Greenland. The final objective was to report the occurrence of potential zoonotic bacteria present in the bacterial community of faeces (as an indicator for intestinal reservoirs for zoonoses) from muskoxen of Northeast Greenland, Norway and Northwest Greenland.

## 2. Materials and Methods

### 2.1. Sampling

**Figure 1:**
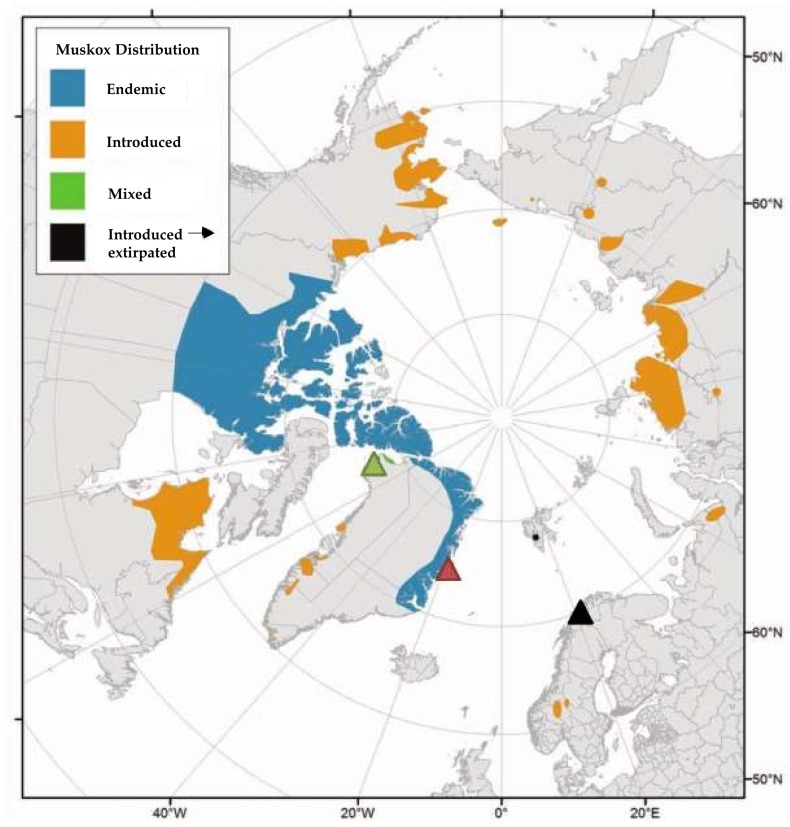
(**a**) Distribution of muskoxen worldwide and indication of sampling sites. Green triangle: Dundas Village (“NWGM,” *n* = 4 faecal samples), red triangle: Zackenberg Research Station (“NEGM,” *n* = 35), black triangle: Tromsø, Ryøya (“NM,” *n* = 3), modified from [1]; (**b**) Muskoxen by the foot of Zackenberg mountain, near Zackenberg Research Station, Photo: Lars Holst Hansen.

In total, 35 adult wild muskoxen (29 females, six males) from the national park of Northeast Greenland (NEGM) were immobilized in September 2013 and September 2015 on the tundra at Zackenberg Research Station (74°28′ N, 20°34′ W) (Figure 1) [23]. Faecal samples were collected directly from the rectum using nitrile laboratory gloves (TouchNTuff^®^ Ansell Healthcare, Iselin, NJ, USA) and prior to insertion of rectal thermometer for anaesthetic monitoring. Age was determined based on horn morphology, in agreement with Olesen and Thing 1984 [24]. Body mass (kg) was determined by lifting the animal on a blanket attached to six luggage scales.

Faecal samples were collected from three adult Norwegian muskoxen (NM) 3 September 2010, immediately after dropping while grazing on patches of grassland and heather in open birch and pine forests, on the Island of Ryøya (69°33′ N, 18°43′ E) outside Tromsø in northern Norway [16] (Figure 1). These animals belonged to UiT: the Arctic University of Norway and were kept for research purposes. This population descends from 25 muskoxen imported in 1969 from East-Greenland, King Christian X Land, in the Kaiser Franz Joseph Fjord and Moskusoksefjorden area.

Four faecal samples were collected from the ground on July 24th within Dundas Village, Thule, North-West Greenland (76°34′ N, 68°50′ V, muskoxen from this area are abbreviated NWGM) (Figure 1). These animals were introduced in the 1980s from an East Greenland muskoxen population from Jameson Land [25]. These samples were, unlike for NEGM and NM, not collected fresh/immediately after dropping (later referred to as “semi-dry” samples in this work). Age of samples is not known but most likely few days <3 months, while: the faeces upon sampling were only semi-dry/still to some degree soft; predominantly dark brown (droppings turn nearly white with time in the high Arctic); they still emitted distinct faecal odour; and the herd had reached the location 3 months earlier and was gone at the time of sampling. However, it cannot be completely ruled out that the samples may originate from less than four individuals.

All samples were frozen immediately after collection and stored at −20 °C before further analysis. Since sampling was carried out differently for NWGM samples compared to NEGM and NM samples, only limited comparisons between the Western Greenland and other samples could be made.

Research permits were granted by the Greenlandic government (J. No. G13-029 and G15-019, in 2013 and 2015, respectively) and by the Greenlandic police (J. No 55se-50190-00153-15, in 2013).

### 2.2. Extraction and Library Construction

The outermost surface (>2 mm) of faecal samples was removed via sterile scalpel before 0.25 g of faecal samples underwent DNA extraction using a QIAamp PowerFecal DNA kit (Qiagen, Hilden, Germany) as per manufacturer’s instructions. All extracted samples were diluted to 3 ng/uL to standardize between samples for further processing. Bacterial metabarcoding was performed on the V3-V4 region of the 16S rRNA region, using the 341F (5′-CCTAYGGGRBGCASCAG-3′) and reverse 806R (5′-GGACTACNNGGGTATCTAAT-3′) primers [26]. Internal tags ranging between 6 and 8 base pairs were added to primers to increase the number of samples that could be multiplexed per library. PCR reactions consisted of: 1 µL of DNA template, 2.5 µL of ×10 buffer, 2.5 µL of 25 mM MgCl_2_ and 1.5 µL of the forward and reverse primers (at 10 mM µL^−1^), 1 µL of BSA (20 mg/mL), 0.2 µL of sNTPs (25 mM) and 0.2 µL of AmpliTaq Gold (Applied Biosystems; Thermo Fisher Scientific, Waltham, MA, USA). Cycling conditions consisted of an initial extension of 5 min at 95 °C, followed by 23 cycles of 95 °C for 15 s, 55 °C for 30 s and 72 °C for 40 s, with a final extension of 72 °C for 4 min also applied. DNA quality of PCR products was checked using the Qbit 2.0 fluorimeter (Thermo Fisher Scientific).

Samples were pooled for library build and purified using a QIAQUICK PCR Purification Kit (QIAGEN, Hilden, Germany). Samples were subsequently prepared for sequencing using a TruSeq DNA PCR-Free Library Preparation Kit (Illumina, San Diego, CA, USA) as per manufacturer’s instructions. Libraries were checked using the 2100 Bioanalyzer (Agilent Technologies, Palo Alto, CA, USA). Final libraries underwent a purification step using AMPure XP magnetic beads in a 1:1.6 ratio of beads to library products (Beckman Coulter; Fisher Scientific, Hampton, NH, USA) and were sequenced on an Illumina MiSeq 2500 (Illumina, San Diego, CA, USA), with 250 base pair paired-end sequencing performed at the National High-Throughput Sequencing Centre of Denmark (Copenhagen, Denmark).

### 2.3. Bioinformatic Analysis

Paired ends were joined using VSEARCH (version 2.3.4) [27] and denoised, with a single base pair per read being the maximum permissible error rate. Using the internal tags, individual libraries were demultiplexed using a custom script that removes adapters, primers and the internal tags using CutAdapt (v1.9.1) [28]. Reads shorter than 400 base pairs were also excised. Reads were only assigned to samples with exact matches of both forward and reverse reads, to conservatively assign reads to samples despite tag jumping [29]. Reads were initially clustered into Operational Taxonomic Units (OTUs) based on a 97% similarity level using the UCLUST algorithm (version 1.2.22) [30]. Chimeras were checked using the reference-based chimera checking algorithm of VSEARCH [27], while using the SILVA database [31] as a reference. Data was subsequently analysed in QIIME (v1.9.1) [32], with OTUs assigned taxonomically using the Ribosomal Database Project (RDP) classifier and the SILVA database and finally reads were deposited in the Sequence Read Archive (Bioproject: PRJNA473762). An OTU table was constructed with chimeras and singletons excluded. A total of 3,393,427 reads were left after denoising, with a minimum read length of 400 base pairs and median length 407 base pairs. Samples ranged from 10,043 and 59,992 reads per sample and were subsequently rarefied to an equal sampling depth of 10,000 reads per sample. After rarefaction, reads were assigned to a total of 3633 unique OTUs spanning 313 different genera, with 0.18% of the OTUs were completely unassigned to any taxonomic level (Spreadsheet S1).

### 2.4. Statistical Analyses

All statistical analyses were performed within *R* (v1.0.143) [33], using the packages Vegan (v2.4.6) [34] and MVABUND (v3.13.1) [35] for analyses and ggplot (v2.2.1) [36] for data visualization.

For our first objective, the physiological and temporal factors regulating the gut bacterial community of the NEGM muskox was assessed by multivariate generalized linear modelling and analysis of variance (MGLM-ANOVA). Each OTU was treated as an explanatory variable and a generalized linear model (GLM) was fitted using a negative binomial distribution. Sex, body mass and year of sampling were used as explanatory variables in these models. This was performed using the manyglm, and ANOVA functions within the MVABUND package of *R*. *p*-values were calculated using 500 resampling iterations via PIT-trap resampling. In order to visualize the community data, a Bray-Curtis similarity matrix was constructed and non-metric multidimensional scaling was performed using the monoMDS function of Vegan. GLM was also performed to test the effects of body mass, sex and year of sampling on OTU richness within these East Greenland samples, also using the negative binomial distribution.

For our second objective, the effect on the microbial community by location and population was studied by comparing the microbial compositions of NEGM, NWGM and NM. As before, MGLM-ANOVA was used to test for compositional effects on the community data using location as the sole explanatory factor, however due the differences in sample collection (fresh vs. delayed/semi-dried faecal collection) the analysis was rerun without the NWGM samples. OTU richness was also investigated as before, with log-normalized OTU richness undergoing GLM to test the effect of location.

Lastly, OTUs were screened for generally accepted zoonoses. Genera were subsequently compared to a list of generally accepted zoonotic diseases described by Acha and Szyfres 2001 [37]. Acknowledging the ability for many bacteria to infect people from animal sources given the right conditions, we also compared our results to a list of bacteria associated with human infections by Paul 2012 [38].

## 3. Results

### 3.1. Physiological and Temporal Effects

**Figure 2:**
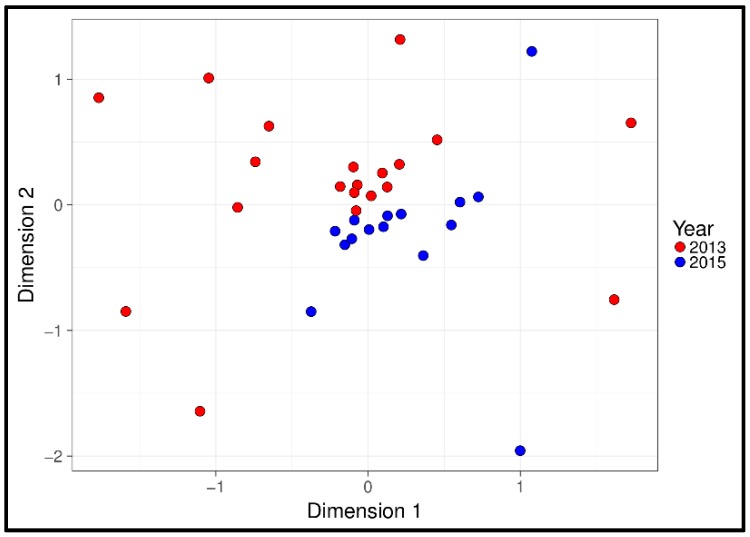
Non-metric multidimensional scaling plot of the bacterial microbiome from muskox faecal samples, produced by 16S DNA metabarcoding. Colour represents the year of sampling, demonstrating clear separation between sampling times.

For NEGM, MGLM-ANOVA identified the factors year of sampling and the animal’s body mass as significantly correlated to faecal bacterial composition (Figure 2, Table 1). There was, however, no effect of the animals’ sex and composition of the bacterial community. GLM revealed no significant variation in OTU richness with either year of sampling or body mass, nor did the sex of the host effect the bacterial richness.

### 3.2. Effect of Location

When NWGM were included in the MGLM-ANOVA, geographical location of the muskoxen was significantly correlated to bacterial composition, whilst GLM revealed a strong effect on OTU richness (Table 1). However, given the differences in sampling of NWGM, these samples were excluded and analyses rerun. Again, a significant effect of geographic location was identified on the bacterial composition, whilst it also had a significant effect on OTU richness (Table 1).

**Figure 3:**
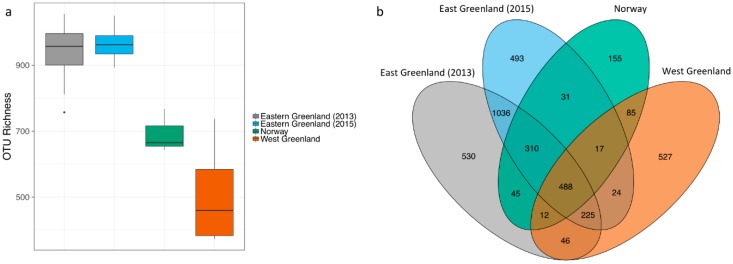
(**a**) Boxplot of OTU richness between NEGM, NM and NWGM. Line within boxes presents the median; (**b**) Venn diagram depicting the shared number of OTUs between North East Greenland muskoxen (NEGM), Norwegian muskoxen (NM) and North West Greenland muskoxen (NWGM).

OTU richness was generally greater for NEGM than NM, average richness = 950 unique OTUs per sample (SD = 64) versus average richness per sample = 692, (SD = 66), respectively. The NM samples collectively presented 1143 different OTUs (*n* = 3) vs. 3257 by NEGM (*n* = 35, Figure 3) and they shared 903 OTUs between them (representing 28% of the NEGM total OTUs and 79% of the NM). Mean richness per sample was lower for NWGM (not fresh samples) than NM and NEGM with an average richness = 507 and there was greater variation between samples (SD = 169). Total OTU richness of NWGM samples was slightly higher than NM with 1424 unique OTUs, whereof 897 (63%) were shared with NEGM and NM. OTU richness generally corresponded with the differences in sample size, with the lowest overall richness presented by the smallest sample size (NM) and vice versa.

**Figure 4:**
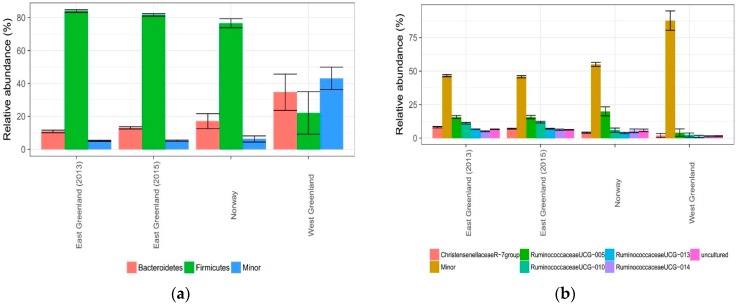
Taxonomic bacterial composition by relative abundance of (**a**) phylum; (**b**) genus.

Firmicutes dominated the bacterial community at the phylum level within the NEGM and NM, with a mean relative abundance of 83% (SD = 3.54%) and 77% (SD = 4.7%) respectively (Figure 4).

**Figure 5:**
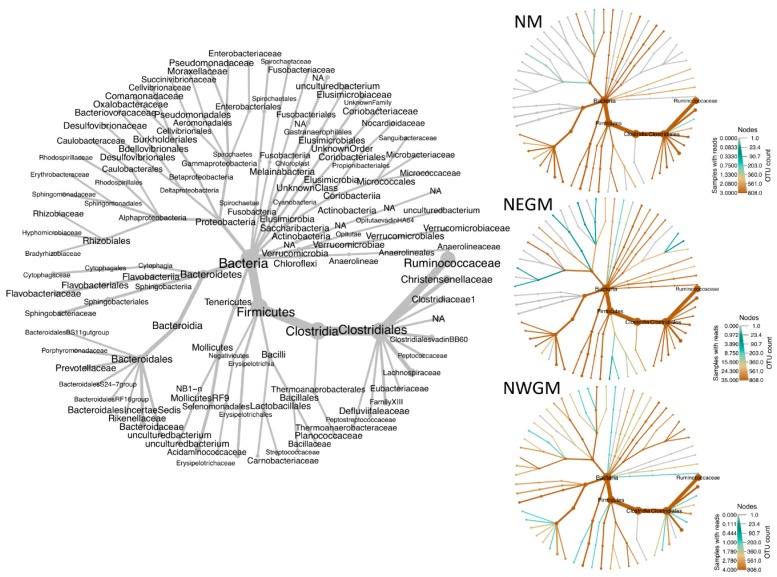
Heat map trees of the bacterial community detected by 16S-rDNA metagenomic sequencing from faecal samples of Norwegian muskoxen (NM), Northeast Greenland muskoxen (NEGM) and Northwest Greenland muskoxen (NWGM). (**Left**) Taxonomic assignments of branches, kingdom—family level; (**Right**) heat map trees for each location sampled. Note the color-coded density scales on the right.

NWGM was more heterogeneous in taxonomic composition at phylum level but Firmicutes and Bacteroidetes remained dominant, though the relative abundance of Firmicutes had reduced dramatically and unlike NM and NEGM, Bacteroidetes dominated over Firmicutes (Figure 4 and Figure 5). Clostridia was the dominant class for both NEGM and NM with a mean relative abundance of 82% (SD = 3.6%) and 75% (SD = 4.5%), respectively. Likewise, the dominant order corresponded between NEGM and NM, with Clostridiales comprising 82% (SD = 3.6%) and 75% (SD = 4.5%) of the community respectively. The dominant family for NEGM and NM was Ruminococcaceae with a relative abundance of 60% (SD = 4.1%) and 51% (SD =1.7%), respectively, representing the majority of the Clostridiales class and Firmicutes phylum. Every other family was below 10% in relative abundance for NEGM and below 14% for NM (Spreadsheet S1).

NWGM samples were substantially different from NM and NEGM below phylum level, with a more heterogeneous presentation of families dominated by Flavobacteriaceae by 17% (SD = 19.3%), Ruminococcaceae by 13% (SD = 14.9%) and Sphingobacteriaceae by 10% (SD = 8%) and greater variation between samples.

### 3.3. Potential Zoonotic Bacteria

Thirteen genera of notable zoonotic potential were detected, including *Escherichia* and/or *Shigella*, *Erysipelothrix*, *Clostridium*, *Bacteroides*, *Bacillus*, *Actinomyces*, *Enterobacter*, *Fusobacterium*, *Pseudomonas*, *Rhodococcus*, *Streptococcus* and *Yersinia*. Species level assignments were generally not determined by the metabarcoding approach, that is, using the V2-V3 16S rRNA region as reference. Table 2 summarizes the potential zoonotic species that these genera comprise. In accordance with its larger sample size, 10 out of 13 zoonotic genera were identified in NEGM samples, yet nine were found in the NWGM samples and seven in the NM samples despite their limited small sizes. The presence of the genera varied considerably between muskoxen groups. *Erysipelothrix*, *Actinomyces* and *Fusobacterium* were for example identified in NEGM but not in NM or NWGM. *Yersinia* and *Rhodococcus* were on the other hand not found in NEGM but only in NWGM and NM, respectively (Table 2). The most frequently identified zoonotic genus across samples was *Clostridium* (35/42) followed by *Escherichia*/*Shigella* (28/42) and *Bacillus* (21/42). For *Clostridium*, 33 out of 35 total occurrences were identified from NEGM. 2/4 NWGM presented *Clostridium* OTUs and no Clostridia were found in the samples of NM. For *Escherichia*/*Shigella* 25/35 were identified in NEGM, 2/4 from NWGM and 3/3 from NM samples were positive. Lastly, for *Bacillus*, 16 out of 35 total occurrences originated from NEGM; 1/3 NM samples were positive for *Bacillus* OTUs and 4/4 NWGM were positive (Table 2).

Moreover, OTUs were assigned to 41 genera associated with human infections (not generally classified as “zoonoses”) were identified by the SILVA database (Table S1).

## 4. Discussion

### 4.1. Physiological and Temporal Effects

The bacterial composition of NEGM were tested against three parameters: body mass, year of sampling and sex and significant effects of body mass and year of sampling were found. Changes in body mass could be associated with the animal’s age and age-related development of the gut microbial composition in muskoxen. Jami et al. [42] found that it takes at least two years for the bovine gut bacterial community to mature and similar findings have been shown for humans [43,44]. Here, the muskoxen were determined as older than two years old but it remains unknown, when, the gut microbial community reaches maturity in high Arctic muskoxen. Changes in gut microbial community associated with changes in weight could be a reflection of bacterial community influence on the animal’s energy metabolism [45], or they could simply be a result of consuming more energy-rich pastures. Gut bacterial composition has been linked to the health of cows [45,46] and being malnourished has significant effects on the gut microbial communities of many animals [47,48,49]. Therefore, the observed changes in the muskox gut bacteria might also be driven by- or impacting upon fitness.

The effect of temporal variation on the gut bacterial community is a feature shared with other animals [50,51,52] and likely reflects changes to their surrounding ecosystem such as feed availability [53,54]. For example, the feed intake by reindeer fluctuates by a factor of 3–4 times as much in summer compared to winter, with significant effects on their gut bacterial community [49,55] and similar findings are reported in muskoxen [56,57,58]. Our samples were collected in the same time of year but could be impacted by inter-annual climate variations, such as prolonged periods of a warm climate. It has been reported that increased temperatures and humidity correlate with changes in gut microbial diversity in cows [59] and is of considerable interest within the muskox given the large environmental changes currently underway in the Arctic. Furthermore, the composition of the gut bacterial community, specifically a high Firmicutes/Bacteroidetes ratio in the rumen, has been found to correlate with higher fat content in milk [45], a factor of high importance for the new-born muskoxen in the high Arctic. This aspect can however not be directly reflected in our results as there are significant variation between ruminal and faecal samples (see more on this below) but it is indicative of potentially broad physiological changes brought about by changes to the gut microbial community.

The phyla Firmicutes and Bacteroidetes dominated the faecal bacterial community of NEGM. Both are effective degraders of complex fibres and they are typical phyla to dominate the faecal microbiome across multiple species and types of digestive tracts, for example, cattle, horses and humans [60,61,62,63]. The principle role of Bacteroidetes is degradation of complex carbohydrates into butyrate in the large intestine. The substrate of Bacteroides is among other cellulose, xylan and pectin but also host-derived gastrointestinal carbohydrates such as mucin [64,65]. Firmicutes were dominated by the order Clostridiales which has been regarded as taking a pivotal role in the degradation and fermentation of cellulose in the large intestine by the secretion and cell-surface incorporation of several well-known cellulases [64]. The dominant family within the Clostridiales was Ruminococcaceae; a family with the ability to degrade otherwise recalcitrant polysaccharides such as complex fibres [66]. Therefore the muskox gut bacterial community seems well adapted to a highly lignified plant diet found within the high Arctic [4,53].

### 4.2. Effect of Location

Location of the muskoxen herds had the greatest effect on both gut bacterial composition as well as richness, even when the dried NWGM faecal samples were excluded. The effects of year of sampling and body mass on the NEGM were relatively small compared to effects of location. Ishaq et al. [67] found similar effects in moose (*Alces alces*) across three Arctic areas; their study however focused on ruminal samples instead of faecal (more on this type of comparison below).

The microbial compositions of NEGM and NM were considerably more similar than NWGM (Figure 4 and Figure 5). Vogtmann et al., 2017 [68] compared the microbiomes of faecal samples frozen immediately after sampling with samples left at ambient temperature for 4 days prior to freezing and found that results were relatively stable. The results of NWGM are nevertheless regarded as most likely being a consequence of the differing sampling technique, with the vast difference between NWGM and NM/NEGM communities far exceeding expected community variation. For example, Firmicutes dominates faecal bacterial communities across mammal species but the Bacteroidetes/Firmicutes ratio was reversed in NWGM and there were increases in aerobic bacteria known to occur in the Arctic environment (Flavobacteriales and Sphingobacteriales), indicating environmental contamination [69,70]. This raises questions about using dried (delayed sampling from dropping) faecal samples in characterizing the gut-associated microbial communities [71,72] and we therefore recommend interpreting microbial community data produced from dried faecal samples cautiously.

Overall, relative abundances between NEGM and NM were comparable. At family level, the relative abundance of Ruminococcaceae was approximately 10% higher in NEGM than NM; and while this could merely be an effect of a small sample size for NM, an increase of fibre intake has been associated with an increase of *Ruminococcus* species in the hindgut of goats [73] and Ruminococcaceae are known as highly effective degraders of recalcitrant polysaccharides [66,74]. An increase in Firmicutes have also been found in faeces of cattle in association with a high-fibre diet [75]; the increase in relative abundance of Firmicutes in NEGM was however only slight compared to NM. These observations could be an indication of a higher fibre-intake of NEGM compared to NM but more studies are needed. Diet is however one of the strongest effectors on the gut microbial community [11,12,13,74,76]. The muskoxen in Northeast Greenland feed mostly on graminoids (approximately 80% of their diet) and shrubs such as *Salix* spp. [4,7], while the diet of the muskoxen on the island Ryøya at the coast of northern Norway is dominated by natural pastures of heather in open birch and pine forest with patches of grassland [16,77]. During wintertime the Norwegian muskoxen on Ryøya are also fed hay to support their forage needs [77]. The climate is warmer and characterized by more precipitation in Tromsø compared to Northeast Greenland [78,79] and Davidson et al. [18] suggest the use of Norwegian muskoxen as early warning sentinels of climate change.

The main difference between NEGM and NM was mean OTU richness per sample, which was significantly lower for NM. All the muskoxen sampled recently originated from, or have been introduced from East Greenlandic population [25] and moreover, genetic diversity is known to be extremely low for muskoxen [80,81]. In evolutionary and genetic terms, this will likely limit the influence of host genotypic variation on the gut microbial community. As discussed above for differences in relative abundances, diet is very likely an important factor in the difference in OTU richness between NEGM and NM. Bacterial richness is generally dependent on microbiota-accessible carbohydrates [82] and there are several studies finding an effect of increased faecal bacterial richness with increased fibre intake across mammal species including ruminants [83,84,85,86,87]. Some studies however fail to detect this association [88,89,90]. Research is on the other hand less ambiguous when it comes the importance of intestinal microbial composition and diversity for dietary efficacy [91,92,93].

Sequencing the V2–V3 region of the 16S gene in the faecal samples from the Ryøya muskoxen (NM) in this present study gave similar results as those reported by Salgado-Flores et al. [16] sequencing the V1–V3 region, with the Firmicutes and Bacteroidetes dominating their faecal bacterial communities at phylum level with approximately 80% and 20% relative abundance, respectively. The *Ruminococcaceae* were the dominant family with a relative abundance of 51% (SD = 1.7) for NM and 61% (SD = 4.1) for NEGM. However, the longer amplicon lengths used in this study also allowed for a greater proportion of the *Ruminococcaceae* to be taxonomically assigned to genus or family, with just 7.4% of the *Ruminococcaceae* OTUs assigned only to family level and between 25–39% in the study performed by Salgado-Flores and colleagues. It also meant that the percentage of reads unassigned to genus level dropped from approximately 55% to 16% in this study.

Lastly, we wish to note that the majority of studies of ruminants have focused directedly on ruminal microbiomes as opposed to faecal, leaving our results not directly comparable to foregut microbial community composition. Based on our DNA-based approach, we cannot know if the template DNA detected originate from viable bacteria and detected OTUs may represent live or dead bacteria from ascending segments of the digestive tract. It has moreover been shown that many of the same taxa are present across the length of the intestinal tract but at variable abundances [94], with for example the Firmicutes/Bacteroidetes ratio shown to significantly increase in faecal- compared to ruminal samples [95]. As such studies have also shown that faecal samples best reflect the bacterial composition of the distal hindgut [96,97].

### 4.3. Potential Zoonotic Bacteria

Due to the limitations of the metabarcoding, species level assignments were not made, however 13 genera generally accepted as zoonoses were identified (Table 2). In addition to these, 41 genera involved in human infections were identified (Table S1).

Muskoxen are hunted and consumed from Midwest to Northwest Greenland and certain areas of Mideast Greenland. Norwegian muskoxen are generally protected and only very rarely consumed, with the muskoxen of Ryøya being used solely for research and not consumed. Muskoxen have recently been drawn into focus because of the commercial slaughter in West Greenland and the findings of zoonotic *Giardia duodenalis* assemblage A (parasite/protozoan) in muskoxen herds across the Arctic [98,99]. A severe case of Q-fever (*Coxiella burnetii*) in a Greenlandic man associated with muskoxen has been reported [21], whilst they also serve as hosts for parasitic zoonoses such as *Echinococcus* spp. [100,101]. *Coxiella* was not detected in this study but placental secretions or serology may be better means for its detection [102]. Other examples of zoonoses that have been reported as being associated with muskoxen elsewhere, are: *Erysipelothrix rhusiopathiae*, *Chlamydophila* spp., *Brucella suis* biovar 4 and *Yersinia pseudotuberculosis* [103,104,105,106], with *Erysipelothrix* and *Yersinia*-assigned OTUs detected in this work.

The greatest risk of infection in the case of these muskoxen would likely arise from contact or ingestion of with water contaminated by faeces, or edible tissues likewise contaminated with intestinal contents. Standard hygienic measures in relation to butchering and handling will be effective in terms of food safety, though feasibility of its practical application in the current Arctic situation must be taken into consideration. Another considerable risk of infection from zoonoses is the contamination of fresh water from carcasses, such as in the case of *Bacillus anthracis*, *Pseudomonas mallei* and *Yersinia* spp. (Table 2).

It should be noted that the zoonotic genera identified here have variable zoonotic potential within the muskoxen (Table 2). It is for example less likely that they carry typically zoonotic *Escherichia* species/strains (such as verotoxin producing *E. coli*, VTEC) [107] compared to other relatively avirulent strains. Although zoonotic *E. coli* O:157 have been found in wildlife, studies have failed to detect significant reservoirs in northern mammalian wildlife [108,109]. Furthermore, *Escherichia* is a normal bacterial inhabitant of the intestinal flora of mammals and a genus which holds several species, where only few, like certain *E. coli* types, are regarded as zoonotic and this species itself comprises an extreme degree of diversity of which only a handful are regarded zoonotic [110,111]. Other more likely zoonoses to take reservoir in these muskoxen are *E. rhusiopathiae*, *Y. pseudotuberculosis*, *Clostridium difficile/perfringens*, *Bacillus cereus*, *Pasteurella multocida* and *Mycobacterium bovis* since these occur in (wild) animal reservoirs [37,112,113,114] and have been specifically found in Arctic species (incl. muskoxen) [105,115,116]; and/or can survive in the environment for prolonged periods of time [37,117,118,119,120,121].

Some zoonotic bacteria also present a serious health threat to the muskoxen, such as *C. burnetii* (causing abortions among other), *E. rhusiopathiae* and *Y. pseudotuberculosis*. The latter two have caused mass mortality of muskoxen. Recently, *E. rhusiopathiae* was confirmed from carcasses of a mass mortality event of ca. 150 muskoxen in Canada [105] but it has also been implicated in other mortality events between 2009–2013 with occurrences stretching across >200,000 km^2^. Kutz et al. [105] found that the mass die offs were compatible with the introduction of a new strain of the pathogen to naïve populations, underscoring the vulnerability of the muskoxen in a changing Arctic and that *E. rhusiopathiae* could be implicated in declining populations of muskoxen in Canada. Events of pathogen introduction to naïve populations are expected to increase with climate change [18] and temperature increases 2–3 degrees above average were recorded prior to at least two outbreaks of *E. rhusiopathiae* on Victoria Island, Canada and non-specific pneumonias have been related to high temperatures in Norway and in captivity [116,122]. The low genetic diversity found within modern muskoxen populations includes a low variability of the Major Histocompatibility Complex (MHC) genes [80,81,123] that could negatively influence their immune responses. Interestingly, both of the mass die-off associated *Yersinia* and *Erysipelothrix* were identified in the current study. Whether these genera present pathogenic and/or zoonotic species is unknown but provides some insight into the likelihood of these muskoxen as carriers and animal health related risks including mass die-offs. In spite of the small sample sizes of NM and NWGM, *Yersinia* was only identified in NWGM and *Erysipelothrix* in NM only; this again underscores the effect of the habitat on the microbial gut community and thereby zoonotic potential.

The detection of genera such as *Rhodococcus*, detected in NWGM only, may arise on behalf of delayed sampling from dropping, which may have led to contamination of environmental bacteria and changes in the bacterial community. *Rhodococcus* is correspondingly a common soil bacterium and obligate aerobe. It may however also have been a transient member of the gut microbial community and (potentially its fragmented DNA) passed with the feces [124].

More studies that permit the identification of species and strains where relevant, are needed to further elucidate this subject and inform hunters and wildlife biologists about zoonotic risk and wildlife health.

## 5. Conclusions

Muskoxen have diverse gut bacterial communities dominated by the phylum Firmicutes. Their gut bacterial communities are affected by changes in the host habitat and bacterial composition is related to a broad range of physiological functions. Location had the largest measured effect on the gut community, whilst there was significant community variation within populations (with body mass and inter-annual variation effecting both richness and composition) but the effects of these were comparatively small. Sampling technique (fresh vs. dried) however, most probably, had the greatest effect on the community composition. A total of 13 genera comprising generally accepted zoonotic bacterial species were identified from the faecal samples and muskoxen may be a substantial reservoir of zoonoses. However, it remains unknown whether these genera represent a significant risk of zoonotic infection for humans and further molecular work identifying zoonotic bacteria to species level is needed.

## Figures and Tables

**Table 1:** Statistical results. Testing the correlation of year of sampling (Year), Sex and weight (Body mass, kg) with OTU richness (GLM) and bacterial composition (MGLM-ANOVA), respectively, in faeces from 3 muskoxen populations. Because of biased sampling procedure, the statistical tests were performed ± Northwest Greenland muskoxen (NWGM).

NEGM Only (*n* = 35), Biotic and Temporal Effects					
MGLM-ANOVA (Composition)					
Variable	Df	Deviance	*p*-value		
Year	33	5584	0.002		
Sex	32	3421	0.080		
Body mass (kg)	31	3951	0.048		
GLM (OTU Richness)					
Variable	Df	Deviance	AIC	*p*-value	
Year	1	120,604	392.4	0.362	
Sex	1	119,037	391.94	0.541	
Body mass (kg)	1	117,833	391.58	0.895	
Location Effects, All Groups (±NWGM)					
MGLM-ANOVA (Composition)					
	Variable	Df	Deviance	*p*-value	
Including NWGM	Location	35	3.161	0.002	
Excluding NWGM	Location	35	3.161	0.002	
GLM (OTU Richness)					
	Variable	Df	Deviance	AIC	*p*-value
Including NWGM	Location	2	2.25	64.134	>0.001
Excluding NWGM	Location	2	0.464	−55.579	>0.001

**Table 2:** Zoonotic genera present in the muskoxen faecal samples collected from Northeast Greenland muskoxen (NEGM), West Greenland muskoxen (NWGM) and Norwegian muskoxen (NM). Includes examples of known zoonotic species within each genus as well as comments of risk and epidemiology/epizootiology.

Genera	Location/No. Animals			Zoonotic Species Examples	Disease in Man	Risk and Epizootiology
	NEGM	NM	NWGM			
*Actinomyces*	4/35	0/3	0/4	*A. bovis*; *A. pyogenes*	Abscesses; chronic bronchopneumonia; sepsis; endocarditis.	Rare infection in people. Worldwide distribution. Cows are the main carrier.
*Bacillus*	16/35	1/3	4/4	*B. anthracis*	“Anthrax”	For man, the source of infection is always infected animals, contaminated animal products or spores from originating from infected animals. (Cutaneous) Infection is for example, known to occur when skinning or butchering an animal or by contact with infected leather, pelts, wool, or fur. Broken dermis favours transmission. (Gastrointestinal) infection can be acquired from consumption of domestic and wild animals. Animals mainly become infected by ingestion of pasture or water that have been contaminated with spores—typically in the vicinity of anthrax-infected carcasses as dead and dying animals are the vessel of high rate of replication of *B. anthracis*. The bacilli sporulate if the carcass is opened and they contaminate the surrounding environment, leading to new infections in particularly grazing animals. Infected animals and especially birds can effectively transport the infection to other areas. Transmission between humans is a rare form of infection for humans.
*Bacteroides*	35/35	3/3	3/4	*Bacteroides* sp.; *B. fragilis*; *B. tectus*; *B. ovatus*	Wound infections	Unknown species of *Bacteroides* associated with purulent wound infection from a horse bite. Wound infections with other species of *Bacteroides* associated with cats and dogs.
*Clostridium*	33/35	1/3	1/4	*C. difficile*; *C. perfringens*	Gastroenteritis	Both types are regarded as potential zoonoses. Infection is acquired from the environment, from contact or ingestion of contaminated meat/animals/animal products [39].
*Erysipelothrix*	15/35	0/3	0/4		Dermatitis; septic arthritis; endocarditis; sepsis	The course of disease usually only lasts for up to four weeks but sepsis with potential subsequent endocarditis can occur. Distributed Worldwide in a wide range of species incl. domestic animals, for example, swine and ruminants and frequently associated with fish. Many animals carry the disease non-symptomatically. People can acquire the infection through handling of infected animal products.
*Escherichia/Shigella*	23/35	3/3	2/4	*E. coli* (VTEC and EHEC) ^1^	Enteritis	Infection through faecal-oral route, commonly from contaminated animal products. Airborne transmission via dust is also possible. EHEC infections in humans often associated with cattle which are considered a reservoir species. VTEC infections associated with both cattle, sheep among other.
*Fusobacterium*	10/35	0/3	0/4	*F. nucleatum*; *F. necrophorum* (*not confirmed*)	Wound infections	Infection risk associated biting events or contamination of open skin lesions or through mucus membranes. Previously predominantly associated with dog bite.
*Mycobacterium*	2/35	0/3	2/4	Principally *M. bovis* in terms of zoonoses; *M. avium* subsp. *paratuberculosis*; *M. simiae*; *M. kansasii*; *M. ulcerans*	Pulmonary and extrapulmonary forms. The latter affecting glands, bones and joints, meninges, urinary tracts and more.	*Mycobacterium* spp. vary with host-species but zoonotic *Mycobacterium* spp. can generally infect a wide range of animal species and are considered to be distributed virtually Worldwide. These bacteria can be difficult to kill in the environment as they are resistant to many common disinfectants as well as desiccation. Pasteurization of milk has however reduced the incidences of zoonotically acquired infections. Infection can however also be acquired through inhalation. Main reservoir of *M. bovis* is cattle. The infection with *M. bovis* comes from animal sources since human to human transmission is considered rare.
*Pasteurella*	0/35	1/3	0/4	*P. multocida*	Disease often associated with bite wounds; abscesses; cellulitis; meningitis; septic arthritis; osteomyelitis; respiratory tract disease; sepsis and endocarditis are rare	Infection is typically acquired through bite wounds but can also occur through inhalation and the digestive tract. Cats and dogs are frequent carriers but cattle and sheep and other also present important asymptomatic reservoirs. Many wild animals are also asymptomatic carriers and outbreaks in wildlife occur occasionally. *Pasteurella* is believed only to survive shortly in the environment and animals therefore play a key role in the epidemiology and infection of *Pasteurella* in people. Infection between humans are also thought to occur.
*Rhodococcus* (syn. Coryne-bacterium)	0/35	0/3	2/4	*R. equi*	A rare disease in humans and often associated with immuno-suppressive states; but neglected reporting is suspected; granulomatous and suppurative lung disease; Pulmonary (most prevalent form) and extra-pulmonary form including osteomyelitis; cachexia; bloody diarrhoea; abscessation and other.	A saprophyte reported Worldwide which replicates effectively in faeces from herbivores such as goats, sheep, cows, deer, horses, cats and dogs. Often isolated from horses and a significant disease of foals. Infection in humans occurs through inhalation of the pathogen, often mediated by dust-particles or through infection of for example, infected sputum. Acetic acid in faeces is believed to favour effective replication of *Rhodococcus* in faeces. The main reservoir is as such soil exposed to faeces from herbivores which shed high amount of acetic acid in their faeces [40,41].
*Streptococcus*	11/35	3/3	2/4	*S. bovis*; *S. suis*; *S. zooepidemicus* (Lancefield group D and C respectively)	*S. suis*: meningitis; arthritis and endophthalmitis; *S. zooepidemicus*: pneumonia; endocarditis; meningitis; pericarditis; exudative pharyngitis; tonsillitis.	Ingestion of for example, raw milk or pork meat and handling of infected animals are related risks. *S. zooepidemicus* is a commensal of the skin on, upper respiratory tract and in the tonsils of various animal species. It causes multiples diseases in horses and is implicated in mastitis of cattle. *S. suis* is a highly occupational disease in relation to slaughterhouses and butchering and believed most often to be acquired through the skin.
*Yersinia*	0/35	0/3	2/4	*Y. enterocolitica*	Enteritis and acute diarrhoea; reactive arthritis; nodular erythema	Worldwide distribution and isolated broadly in the environment: from animals, food and water. Can occur sporadically or as epidemics. Faecal-oral route of transmission from other people or from contaminated animal products. Occupation involving swine, consumption of pork and milk products are associated with zoonotic transmission to people.

^1^ VTEC = Vero-toxin Producing *E. coli*; EHEC = Enterohemorrhagic *E. coli.*

## Supplementary Materials

The following are available online at http://www.mdpi.com/2076-2607/6/3/76/s1, Table S1: bacterial genera associated with human infections detected in muskoxen feces. Spreadsheet S1: muskoxen faecal 16S rRNA metabarcoding, data.
