# Supplementary material for: A Comparative Study on the Faecal Bacterial Community and Potential Zoonotic Bacteria of Muskoxen (Ovibos moschatus) in Northeast Greenland, Northwest Greenland and Norway
Source: Microorganisms. 2018 Jul 25;6(3):76. doi: 10.3390/microorganisms6030076 (PMC6164070; doi:10.3390/microorganisms6030076)
Supplement: Supplementary file 1 [file microorganisms-06-00076-s001.zip › microorganisms-317518-Supplementary Materials/Table S1. Bacteria ass. with human infections detected in muskoxen feces.docx]

**Table S1.** Bacterial genera (41) associated with human infections detected in muskoxen feces. Adapted from J. Paul (2012) “A checklist of bacteria associated with infection in humans, in: Oxford textbook of medicine: Infection”.

| **Genus** | **Species implicated in human infections** | **Presentaton of Infection** | **Comments** |
| --- | --- | --- | --- |
| ***Achromobacter (Alcaligenes)*** | *A. denitrificans* | Septicaemia, CAPD peritonitis, pneumonia, ear infection, pulmonary infection in cystic fibrosis, keratitis, vascular line sepsis |  |
|  | *A. insolitus* |  |  |
|  | *A. piechaudii* |  |  |
|  | *A. ruhlandii* |  |  |
|  | *A. spanius* |  |  |
|  | *A. xylosoxidans* |  |  |
| ***Arthrobacter*** | *A. albus* | UTI, bacteraemia, skin infection | *Arthrobacter* sp. has been implicated in Whipple’s syndrome, a disease usually associated with *Tropheryma whipplei* |
|  | *A. creatinolyticus* |  |  |
|  | *A. cumminsii* |  |  |
|  | *A. luteolus* |  |  |
|  | *A. oxydans* |  |  |
|  | *A. scleromae* |  |  |
|  | *A. woluwensis* |  |  |
| ***Atopobium*** | *A. minutum (Lactobacillus minutus)* | UTI, dental abscesses, pelvic abscesses, wound infection | Isolates from periodontal sites suggest possible role in periodontal disease |
|  | *A. parvulum (Streptococcus parvulus)* |  |  |
|  | *A. rimae (Lactobacillus rimae)* |  |  |
|  | *A. vaginae* | Bacterial vaginosis |  |
| ***Bradyrhizobium*** | *B. elkanii* | Detected in tissue from aortic aneurysm | Potential role as pathogen uncertain |
| ***Brevundimonas* (*Pseudomonas*)** | *B. diminuta* | Septicaemia, endocarditis |  |
|  | *B. vesicularis* |  |  |
| ***Butyrivibrio*** | *B. fibrisolvens* | Endophthalmitis | From rumina of farm animals |
| ***Cellulomonas*** | *C. denverensis* | Bacteraemia, meningitis, pilonidal abscess, wound infection, homograft valve infection |  |
|  | *C. hominis* (CDC coryneform group A-3) |  |  |
| ***Chryseobacterium* (*Flavobacterium*)** | *C. gleum* | Bacteraemia, abdominal sepsis, vascular catheter sepsis | Susceptibilities vary. Often multiresistant |
|  | *C. indologenes* |  |  |
| ***Collinsella*** | *C. aerofaciens* |  | From faecal flora. Clinical significance is undefined |
| ***Comamonas* (Pseudomonas)** | *C. terrigena* | Bacteraemia, UTI, conjunctivitis, endocarditis, wound infection, abdominal abscess, peritonitis, meningitis | Infections in neutropenic patients. Infections associated with animal bite and exposure to tropical fish |
|  | *C. testosteroni* |  |  |
| ***Corynebacterium*** | *C. accolens* | Septicaemia, peritonitis, UTI, eye infection, wound infection, endocarditis, osteomyelitis, septic arthritis, meningitis, abscesses | More than 40 *Corynebacterium* spp. have been isolated from clinical specimens. For many of them, clinical significance and empirical therapy are poorly defined. Many isolates are susceptible to β-lactams. Multiresistant, vancomycin-susceptible isolates of CDC coryneform group G-2, *C. jeikeium* and *C. urealyticum*have been reported. Nosocomial outbreaks have been reported. *Corynebacterium*spp. may be specimen or laboratory contaminants. CDC coryneform groups 1, E, F-1, and G-2 await designation of scientific names |
|  | *C. afermentans* |  |  |
|  | *C. amycolatum* |  |  |
|  | *C. appendicis* |  |  |
|  | *C. argentoratense* |  |  |
|  | *C. atypicum* |  |  |
|  | *C. aurimucosum (C. nigricans)* |  |  |
|  | *C. auris* |  |  |
|  | *C. bovis* |  |  |
|  | *C. confusum* |  |  |
|  | *C. coyleae* |  |  |
|  | *C. durum* |  |  |
|  | *C. falsenii* |  |  |
|  | *C. freneyi* |  |  |
|  | *C. glucuronolyticum* |  |  |
|  | *C. imitans* |  |  |
|  | *C. jeikeium* |  |  |
|  | *C. kroppenstedtii* |  |  |
|  | *C. kutscheri* |  |  |
|  | *C. lipophilum* |  |  |
|  | *C. macginleyi* |  |  |
|  | *C. matruchotii* |  |  |
|  | *C. mucifaciens* |  |  |
|  | *C. pilosum* |  |  |
|  | *C. propinquum* |  |  |
|  | *C. renale* |  |  |
|  | *C. resistens* |  |  |
|  | *C. riegelii* |  |  |
|  | *C. sanguinis* |  |  |
|  | *C. singulare* |  |  |
|  | *C. striatum* |  |  |
|  | *C. sundsvallense* |  |  |
|  | *C. thomssenii* |  |  |
|  | *C. tuberculostearicum* |  |  |
|  | *C. tuscaniense* |  |  |
|  | *C. urealyticum* |  |  |
|  | *C. xerosis* |  |  |
|  | *C. diphtheriae* | Diphtheria, cutaneous infection | Toxigenic infection requires treatment with antitoxin |
|  | *C. minutissimum* | Erythrasma, bacteraemia, endocarditis | Role as an agent of erythrasma is poorly defined |
|  | *C. mycetoides* | Tropical ulcer, septicaemia |  |
|  | *C. pseudodiphtheriticum* | UTI, endocarditis, lymphadenopathy, necrotizing tracheitis |  |
|  | *C. pseudotuberculosis* | Lymphadenitis, pulmonary infection | Associated with sheep contact. May require drainage or excision |
|  | *C. ulcerans* | Diphtheria-like disease, pharyngitis | Toxigenic infection requires treatment with antitoxin |
|  | *C. vitaeruminis* | Associated with aortic aneurysm | Role as pathogen uncertain |
| ***Desulfovibrio*** | *D. desulfuricans* | Bacteraemia, liver abscess |  |
|  | *D. vulgaris* |  |  |
|  | ‘*D. fairfieldensis*’ | Cultured from urine of patient with UTI and meningoencephalitis | Proposed name does not have standing in nomenclature |
| ***Dialister*** | *D. invisus* | Periodontitis, endodontic infection, bacteraemia |  |
|  | *D. micraerophilus* |  |  |
|  | *D. pneumosintes* |  |  |
|  | *D. propionicifaciens* |  |  |
| ***Dietzia*** | *D. maris* | Prosthetic hip infection, bacteraemia | Papillomatosis has been associated with ‘*Dietzia* strain X’ |
| ***Empedobacter*** | *E. brevis* (*Flavobacterium breve*) | Endophthalmitis, bacteraemia, UTI | Carbapenem-resistant |
| ***Flavobacterium*** | *F. mizutaii* (*Sphingobacterium mizutae*) |  |  |
| ***Herbaspirillum*** | *H.* sp. | Associated with aortic aneurism | Detected by 16S gene analysis. Of doubtful clinical significance |
| ***Kurthia*** | *‘K. bessonii’* | Bacteraemia, endocarditis | Not in approved lists of bacterial names |
|  | *K. gibsonii* |  | Isolated from faeces of patients with diarrhoea |
|  | *K. zopfii* |  |  |
| ***Massilia*** | *M. timonae* | Bacteraemia, wound infection |  |
| ***Mesorhizobium*** | *M. amorphae* | Pneumonia |  |
| ***Methylobacterium*** | *M. extorquens* | Bacteraemia, CAPD peritonitis, UTI, septic arthritis | Detected in aortic aneurysm |
|  | *M. mesophilicum* (*Pseudomonas mesophilica*) |  |  |
| ***Mogibacterium*** | *M. diversum* | Endodontic infection |  |
|  | *M. neglectum* |  |  |
| ***Moryella*** | *M. indoligenes* |  |  |
| ***Myroides (Flavobacterium)*** | *M. odoratimimus* | UTI, wound infection | May be multiresistant |
|  | *M. odoratus* |  |  |
| ***Paenibacillus*** | *P. alvei* | Septicaemia, meningitis, pneumonia |  |
|  | *P. macerans* |  |  |
|  | *P. polymyxa* |  |  |
|  | *P. popilliae* |  |  |
| ***Parabacteroides*** | *P. distasonis* | Abscesses |  |
|  | *P. goldsteinii* (*Bacteroides goldsteinii*) |  |  |
|  | *P. merdae* |  |  |
| ***Peptococcus*** | *P. niger* | Abdominal sepsis |  |
| ***Prevotella (Bacteroides)*** | *P. bergensis* | Abscesses, bacteraemia, wound infection, bite infections, genital tract infections, periodontitis, endodontic infection | A genus that includes the well-known former *Bacteroides melaninogenicus*and allied species of anaerobes |
|  | *P. bivia* |  |  |
|  | *P. buccae* |  |  |
|  | *P. buccalis* |  |  |
|  | *P. corporis* |  |  |
|  | *P. dentalis* |  |  |
|  | *P. denticola* |  |  |
|  | *P. disiens* |  |  |
|  | *P. enoeca* |  |  |
|  | *P. heparinolytica* |  |  |
|  | *P. intermedia* |  |  |
|  | *P. loeschii* |  |  |
|  | *P. melaninogenica* |  |  |
|  | *P. multiformis* |  |  |
|  | *P. multisaccharivorax* |  |  |
|  | *P. nigrescens* |  |  |
|  | *P. oralis* |  |  |
|  | *P. oris* |  |  |
|  | *P. oulorum* |  |  |
|  | *P. tannerae* |  |  |
|  | *P. timonensis* |  |  |
|  | *P. veroralis* |  |  |
|  | *P. zoogleoformans* |  |  |
| ***Pseudonocardia*** | *P. autotrophica (Amycolata autotrophica)* |  | Role as pathogen uncertain |
| ***Psychrobacter*** | *P. immobilis* | Meningitis, bacteraemia, eye infection |  |
|  | *P. phenylpyruvicus (Moraxella phenylpyruvica)* |  |  |
| ***Rahnella*** | *R. aquatilis* | UTI, septicaemia | Immunocompromised patients |
| ***Roseomonas*** | *R. cervicalis* | Bacteraemia, wound infection, peritonitis |  |
|  | *R. gilardii ssp.* |  |  |
|  | *gilardii* |  |  |
|  | *R. gilardi ssp.* |  |  |
|  | *rosea* |  |  |
|  | *R. mucosa* |  |  |
| ***Ruminococcus*** | *R. flavefaciens* | Abdominal sepsis, abscesses |  |
|  | *R. hansenii (Streptococcus hansenii)* |  |  |
|  | *R. luti* |  |  |
|  | *R. productus (Peptostreptococcus productus)* |  |  |
| ***Selenomonas*** | *S. artemidis* | Bacteraemia, lung abscess | Malignancy and alcohol abuse reported as risk factors for infection |
|  | *S. dianae* |  |  |
|  | *S. flueggei* |  |  |
|  | *S. infelix* |  |  |
|  | *S. noxia* |  |  |
|  | *S. sputigena* |  |  |
| ***Slackia*** | *S. exigua (Eubacterium exiguum)* | Periodontitis |  |
| ***Sphingobacterium (Flavobacterium)*** | *S. multivorum* | Bacteraemia, pulmonary infection |  |
|  | *S. spiritivorum* |  |  |
|  | *S. thalpophilum* |  |  |
| ***Sphingomonas*** | *S. parapaucimobilis* | Septicaemia, UTI, wound infections, CAPD peritonitis | Nosocomial infections |
|  | *S. paucimobilis (Pseudomonas paucimobilis)* |  |  |
|  | *S. sanguinis (S. sanguis)* |  |  |
|  | *S. yanoikuyae* |  |  |
| ***Streptomyces*** | *S. albus* | Actinomycetoma |  |
|  | *S. anulatus* |  |  |
|  | *‘S. paraguayensis’* |  |  |
|  | *S. somaliensis* |  |  |
|  | *S. bikiniensis* | Bacteraemia, abscess, pericarditis, endocarditis | Treatment options poorly defined |
|  | *S. griseus* |  |  |
| ***Treponema*** | *T. amylovorum* |  | Associated with periodontal disease. Role as potential pathogens unclear |
|  | *T. denticola* |  |  |
|  | *T. lecithinolyticum* |  |  |
|  | *T. maltophilum* |  |  |
|  | *T. medium* |  |  |
|  | *T. parvum* |  |  |
|  | *T. pectinovorum* |  |  |
|  | *T. putidum* |  |  |
|  | *T. scoliodontum* |  |  |
|  | *T. socranskii* |  |  |
|  | *‘T. vincentii’* |  |  |
|  | *‘T. carateum’* | Pinta | Name does not have standing in nomenclature |
|  | *T. minutum* |  | From genital flora. Considered nonpathogenic but have been isolated from genital lesions |
|  | *‘T. phagedenis'’* |  |  |
|  | *‘T. refringens’* |  |  |
|  | *T. pallidum* | Syphilis | *‘T. pallidum endemicum’ is the agent of nonvenereal endemic syphilis* |
|  | *‘T. pallidum endemicum’* |  |  |
|  | *T. pertenue (‘T. pallidum pertenue’)* | Yaws |  |
| ***Veillonella*** | *V. atypical* | Abscesses, bacteraemia |  |
|  | *V. dipsar* |  |  |
|  | *V. montpellierensis* |  |  |
|  | *V. parvula* |  |  |
| ***Xanthomonas*** | *X. campestris* | Bacteraemia |  |
